# Association between residual teeth number in later life and incidence of dementia: A systematic review and meta-analysis

**DOI:** 10.1186/s12877-018-0729-z

**Published:** 2018-02-17

**Authors:** Bumjo Oh, Dong-Hun Han, Kyu-Tae Han, Xibei Liu, Johnson Ukken, Carina Chang, Kiki Dounis, Ji Won Yoo

**Affiliations:** 1 Department of Family Medicine, SMG-SNG Boramae Medical Center, Seoul, South Korea; 2 Department of Preventive & Social Dentistry, Seoul National University School of Dentistry, Seoul, South Korea; 3 Research and Analysis Team, Ilsan National Health Insurance Hospital, Gyeonggi-do, Goyang-si, South Korea; 4 Department of Medicine, University of Arizona College of Medicine, Tucson, AZ USA; 5 University of Nevada School of Medicine, Reno, Nevada USA; 6 Veterans Affairs Southern Nevada Healthcare System, North Las Vegas, Nevada USA; 7 Departmemnt of Internal Medicine, University of Nevada Las Vegas School of Medicine, 1701 W Charleston Blvd. #230, Las Vegas, Nevada 89102 USA

**Keywords:** [MeSH terms]: Elderly, Dementia, Meta-analysis, Teeth

## Abstract

**Background:**

It has been suggested that tooth loss in later life might increase dementia incidence. The objective of this analysis is to systematically review the current evidence on the relationship between the number of remaining teeth and dementia occurrence in later life.

**Methods:**

A search of multiple databases of scientific literature was conducted with relevant parameters for articles published up to March 25th, 2017. Multiple cohort studies that reported the incidence of dementia and residual teeth in later life were found with observation periods ranging from 2.4 to 32 years. Random-effects pooled odds ratios (OR) and 95% confidence intervals (CI) were estimated to examine whether high residual tooth number in later life was associated with a decreased risk of dementia. Heterogeneity was measured by *I*^2^. The Grading of Recommendations Assessment, Development, and Evaluation (GRADE) system was used to assess the overall quality of evidence.

**Results:**

The literature search initially yielded 419 articles and 11 studies (aged 52 to 75 at study enrollment, *n* = 28,894) were finally included for analysis. Compared to the low residual teeth number group, the high residual teeth number group was associated with a decreased risk of dementia by approximately 50% (pooled OR = 0.483; 95% CI 0.315 to 0.740; *p* < 0.001; *I*^2^ = 92.421%). The overall quality of evidence, however, was rated as very low.

**Conclusion:**

Despite limited scientific strength, the current meta-analysis reported that a higher number of residual teeth was associated with having a lower risk of dementia occurrence in later life.

**Electronic supplementary material:**

The online version of this article (10.1186/s12877-018-0729-z) contains supplementary material, which is available to authorized users.

## Background

Dementia is a degenerative neuropsychological syndrome that affects not only a person’s ability to perform everyday activities, but also their cognitive skills including difficulties with memory, language, and problem-solving [1]. These difficulties occur because nerve cells in parts or most of the brain involved in cognitive function have been damaged or destroyed [2]. With the exception of cases of dementia caused by genetic abnormalities, dementia is thought to develop, like other common chronic diseases, as a result of multiple factors rather than a single cause [3]. Dementia is common as it affects 46.8 million older adults worldwide with an annual incidence of 9.9 million [4]. As life expectancy rises across the planet, most sharply in developing countries, the prevalence of dementia is expected to increase as well [4, 5]. Dementia has an enormous social and economic impact on patients, families, and government programs [6]. Thus, there is growing interest in identifying the risk factors for dementia, especially modifiable ones, which may play a pivotal role in preventing or at least delaying the progression of dementia.

Recently, a number of systematic reviews and meta-analyses examining longitudinal studies from Western and Asian countries have focused on the link between the number of teeth and cognitive status [7–9]. Despite the importance of examining the relationship between tooth loss and dementia in later life, only one meta-analysis, which was limited, was recently released [9]. Shen et al. [9] included cross sectional studies that might not accurately estimate dementia incidence and did not include important longitudinal studies in the analysis without readily apparent reasons [10, 11]. In the meta-analysis by Shen et al. [9], neither the process for the literature search nor for assessing the quality of evidence using standardized tools were presented. Therefore, there is an urgent need for a well-designed meta-analysis to examine the relationship between tooth loss and dementia incidence in later life.

## Methods

### Search strategy

A literature search was performed using the keywords “teeth”, “tooth”, “dental”, “tooth loss”, “teeth loss”, “missing teeth”, “oral health”, “dental care”, “elderly”, “later life”, “older adults”, “cognition”, “cognitive impairment”, “cognitive decline”, “dementia”, or in various combinations to identify every original study published in English from PubMed, EMBASE, Medline/Ovid, Cumulative Index to Nursing and Allied Health Literature, Web of Science, and Google Scholar databases up to March 25th, 2017. See Additional file 1 for more detailed information of systematic search strategy. Residual teeth number was defined as the count of natural teeth only and the division between a “high” and a “low” number of teeth in included studies ranged from 11 to 24 teeth (see Table 2). Studies that examined the association between residual teeth number and incidence of dementia in middle-age or older adults were included. Studies depicting high and low residual teeth groups at the time of study completion were given preference. Selected papers were restricted to cohort studies to prevent significant selection bias from cross-sectional studies in determining the incidence of dementia [12].

### Study selection and data extraction

**Fig. 1:**
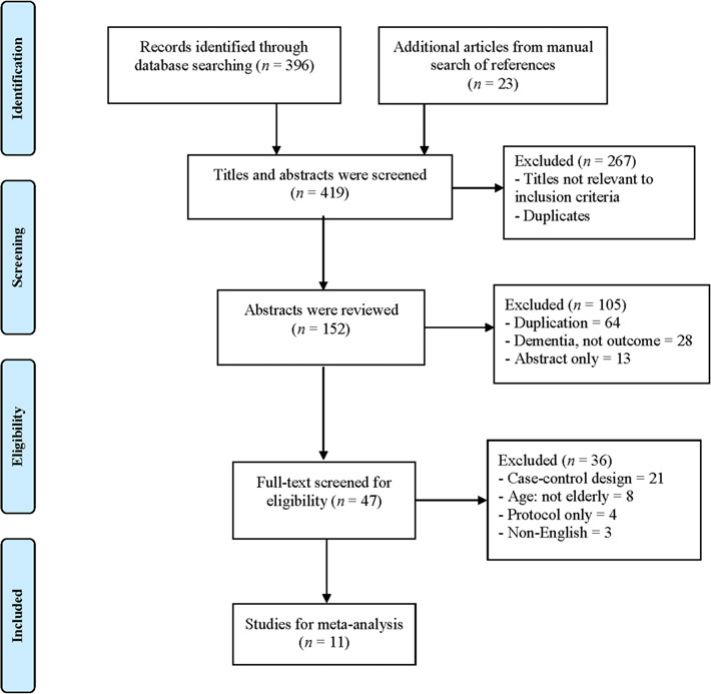
PRIMA Study selection process for meta-analysis

Two authors (X.L and C.C.) independently screened titles and abstracts. Full articles that met the criteria were pooled after an independent review, they extracted the data. Discrepancies were resolved in consultation with two other authors (B.O. and D.H.) every phase. Alternative search approach was performed by manually reviewing the references of eligible articles. Through alternative search, relevant twenty-three studies were added. The high or low teeth number groups were collected from tables and manuscript text in each study. When actual data was not presented in certain studies, two authors (K.H. and J.Y.) contacted the corresponding authors of the studies by either email or phone call in order to access data. Since the data originated from previously published studies, an institutional review board approval was waived. Finally, eleven studies were selected. Figure 1 presents the study selection process in accordance with the Preferred Reporting Items for Systematic Reviews and Meta-Analyses (PRISMA) statement [13]. A summary of each study is shown in Table 1 and Table 2 presents patient specification for each study.

**Table 1:** Studies’ description

Study	Publishing year	Country, setting	Years of observation	Minimum age at enrollment, mean age upon study completion	Study participants at enrollment, *n*	Excluded or lost participants, *n* (%)
Takeuchi et al. [22]	2017	Japan, Hisayama community	5.4	60	1996	430 (21.5%)
Komiyama et al. [23]	2016	Japan, Tsurugaya district community	8	70, 75.2	948	114 (12.0%)
Stewart et al. [24]	2015	Sweden, Gothenburg community	32	70	697	29% in 2000–2001 cohort; 30% in 2005–2006 cohort
Luo et al. [25]	2015	China, Jing’ansi community	3	60, 71.3	3836	3063 (20.1%)
Batty et al. [10]	2013	20 Countries, 215 centers	5	55, 66.2	11,140	1571 (14.1%)
Paganini-Hill et al. [26]	2012	United States, Retirement community	18	52, 80.3	8403	2762 (32.8%)
Yamamoto et al. [27]	2012	Japan, Aichi community	3.7	65	4898	473 (9.6%)
Arrivé et al. [20]	2011	France, Gironde and Dordogne communities	15	66,71.9	447	42 (9.4%)
Kim et al. [11]	2007	Korea, Kwangju community (10/66 Dementia Research Group)	2.4	65, 73.4	919	233 (25.3%)
Stein et al. [21]	2007	United States, Nun community	12	75, 83.0	144	24 (16.7%)
Shimazaki et al. [28]	2001	Japan, 29/30 institutions in Kitakyushu	7	59, 79.7	719	236 (32.8%)

**Table 2:** Study specification: cognitive assessment tool and dementia incidence rate by high/low residual teeth number groups

Study	Publishing year	Cognitive assessment tool	Dementia incidence rate, per 100,000 persons	
			High residual teeth number group	Low residual teeth number group
Takeuchi et al. [22]	2017	DSM-III-R^a^	7391 (≥ 20 teeth remaining)	16,939 (≤ 19 teeth remaining)
Komiyama et al. [23]	2016	MMSE^b^	6417 (≥ 20 teeth remaining)	11,087 (≤ 19 teeth remaining)
Stewart et al. [24]	2015	DSM-III-R^a^	18,304 (≥ 21 teeth remaining)	30,522 (≤ 20 teeth remaining)
Luo et al. [25]	2015	DSM-IV^c^ and MCI^d^	2063 (< 16 missing teeth)	9783 (> 16 missing teeth)
Batty et al. [10]	2013	MMSE^b^	484 (≥ 22 teeth remaining)	1320 (≤ 21 teeth remaining)
Paganini-Hill et al. [26]	2012	MMSE^b^	21,592 (≥ 16 teeth remaining)	19,271 (≤ 15 teeth remaining)
Yamamoto et al. [27]	2012	A standardized questionnaire developed by the Ministry of Health, Labor, and Welfare in Japan	2848 (≥ 20 teeth remaining)	5854 (≤ 19 teeth remaining)
Arrivé et al. [20]	2011	DSM-III-R^a^	31 (< 11 missing teeth)	41 (≥ 11 missing teeth)
Kim et al. [11]	2007	DSM-IV^c^	5556 (≥24 teeth remaining)	9908 (< 24 teeth remaining)
Stein et al. [21]	2007	MMSE^b^	36,468 (unknown)	68,421 (unknown)
Shimazaki et al. [28]	2001	Historical diagnosis information from medical records	13,235 (≥ 20 teeth remaining)	32,739 (≤ 19 teeth remaining)

^a^DSM-III-R = Diagnostic and Statistical Manual of Mental Disorders, Revised, Third Edition

^b^MMSE = Mini-Mental State Examination

^c^DSM-IV = Diagnostic and Statistical Manual of Mental Disorders, Fourth Edition

^d^MCI = Mild Cognitive Impairment

### *Quality assessment*

We used the Grading of Recommendations Assessment, Development, and Evaluation (GRADE) [14] system to assess the overall quality of evidence for each outcome. Five domains (risk of bias, consistency, directness, precision, and publication bias) were considered to assess the overall quality of evidence. The GRADE system rates the quality of evidence as high, moderate, low, and very low [15]. Meta-analysis from observational studies start from a low quality of evidence. The quality of evidence may decrease when there are serious limitations of any of the five domains; therefore, optimal information size (OIS) calculations as an objective measure of imprecision for grading evidence were used. A priori assessment of risk showed an increase by 50% from low residual teeth number group with an alpha = 0.05 and beta = 0.80 compared to the dementia incidence risk from high residual teeth number group [10, 11, 15]. Publication bias was assessed by Egger’s regression analysis, which provides a more objective way to estimate the reliability of results, [16] and by funnel plot for visual inspection of publication bias. Table 3 presented the quality of evidence using the GRADEpro software [17].

**Table 3:** GRADE^a^ quality of evidence

Outcomes	Incidence rate per 100,000 persons		Pooled OR^b^(95% CI^c^, *p*)	№ of participants (studies)	Quality of evidence	Comments
	Low residual tooth number group	High residual tooth number group				
Incidence of dementia	8415 per 100,000 (1209/14,366)	7729 per 100,000 (1123/14,528)	0.483 (0.315 to 0.740, *p* < 0.001)	28,894 (11 cohort studies)	⨁◯◯◯ VERY LOW	Random effects, *I*^2^ = 92.421%

^a^GRADE = Grading of Recommendations Assessment, Development, and Evaluation; ^b^OR = odds ratios; ^c^CI = confidence intervals

### Data synthesis and analysis

Individual study results were combined to calculate the pooled odds ratio (OR) and 95% confidence intervals (CI) using the random effects method [18]. Between-study heterogeneity was assessed using the *I*^2^ static values of 50%, representing an extensive statistical inconsistency. Subgroup analysis was performed to examine the effects of observation period, definition of high residual teeth number group, and study site. Meta-regression analysis was performed to predict whether age, gender, alcohol, smoking, diabetes, hypertension, depression, education attainment, denture use, regular dental care would be associated with the incidence of dementia. Studies were excluded one at a time and meta-analysis findings were individually analyzed against sensitivity analysis, which specifically analyzes to assess the robustness of the review results. For sensitivity analysis on publication bias, the “trim-and-fill” methods proposed by Duval and Tweedie [19] were used to further re-examine publication bias and to estimate corrected pooled ORs. Second order terms as sensitivity analysis of meta-regression results were used. All analyses were performed in Statistical Package for the Social Sciences version 24 (IBM Analytics Inc., Armonk, New York, USA, 2015) and Comprehensive Meta-Analysis version 3 (Biostat Inc., Englewood, New Jersey, USA, 2014). A 2-sided *p*-value < 0.05 was considered statistically significant.

## Results

### Study selection and participants characteristics

A total of 28,642 patients from 11 cohort studies were described in detail in Table 1 [10, 11, 20–28]. Observation periods ranged from 2.4 (Kim et al. [11]) to 32 years (Stewart et al. [24]). Age of enrollment ranged between ages of 52 and 75. High and low residual teeth group participants were 14,366 and 14,528, respectively. Between 9.6% (Yamamoto et al. [27]) and 32.8% (Paganini-Hill et al. [26]) of participants in each study were excluded from study enrollment or lost to follow-up during observation period. Table 2 presents each study’s cognitive assessment tool as well as dementia incidence rates per 100,000 persons by high/low residual teeth tooth groups. Stewart et al. [24] and Paganini-Hill et al. [26] demonstrated the highest rates of dementia incidence as well as the longest observation periods, 32 years and 18 years, respectively.

### Meta-analyses

**Fig. 2:**
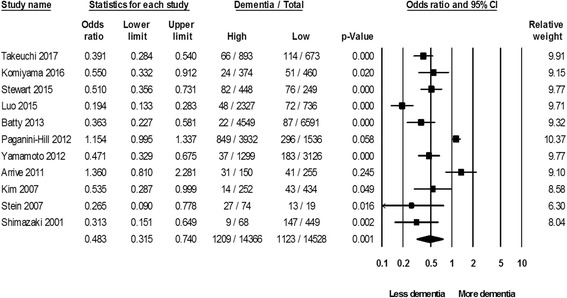
Meta-analysis results: High and low residual teeth number group comparison on dementia occurrence

Figure 2 presents the meta-analysis results, which are the incidence of dementia between high and low residual teeth number groups. High residual teeth number group was associated with a decrease in the dementia occurrence risk (pooled OR = 0.483; 95% CI 0.315–0.740; *p* < .001). Heterogeneity was extensive (Q = 64.722, *p* < .001, *I*^2^ = 92.421%). Pooled incidence rates of dementia in low and high residual teeth group participants were 8415 and 7729 per 100,000 persons, respectively as presented in Table 3.

### *Quality assessment*

**Fig. 3:**
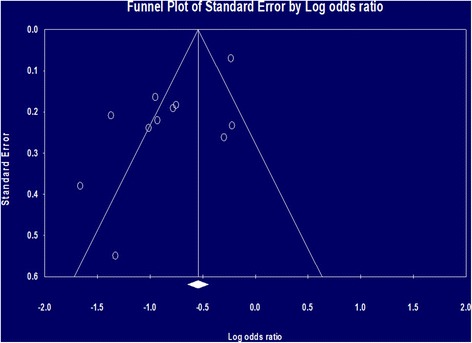
Funnel plot of publication bias

The final quality of evidence was lowered to “very low” because serious limitations were found regarding inconsistency and publication bias of the GRADE [15] system as shown in Table 3. The quality of evidence started low because the analyzed studies were all observational [10, 11, 20–28]. There was evidence of publication bias found by the Egger’s regression test [16] (*p* = .009) and by the funnel plot as shown in Fig. 3. The total number of study participants (28,894) exceeded OIS (1471).

### Subgroup and meta-regression analyses

In the subgroup analysis by length of observation period (≥ 5 years, < 5 years), high residual tooth number group was persistently associated with a decrease in dementia occurrence (OR = 0.545; 95% CI 0.343–0.864; *p* = .010) in ≥5 years [10, 20–24, 26, 28] and (OR = 0.358; 95% CI 0.185–0.693; *p* = .002) in < 5 years [11, 25, 27]. When the high residual tooth number group was defined as ≥20 [10, 22, 23, 27, 28], it was associated with a decrease in dementia occurrence (OR = 0.445; 95% CI 0.378–0.523; *p* < .001). However, this association was diminished (*p* = .264) in other definitions of high residual teeth group [11, 20, 21, 24–26]. In terms of study location, high residual teeth number group was associated with a decrease in dementia occurrence risk in Asian countries [11, 22, 23, 25, 27, 28] (OR = 0.381; 95% CI 0.271–0.536; *p* < .001), but this finding was not observed in Western countries [10, 20, 21, 24, 26] (OR = 0.646; 95% CI 0.364–1.146; *p* = .135).

Table 4 depicts meta-regression analysis results. Old age [10, 11, 20, 21, 23–28] (OR = 1.068; 95% CI 1.007–1.142; *p* < .001), diabetes [10, 22, 24, 25] (OR = 3.542; 95% CI 1.135–4.885; *p* = .037), and hypertension [22, 23, 25, 28] (OR = 3.146; 95% CI 1.071–4.610; *p* = .041) were predictors of dementia incidence. On the other hand, high-level education attainment [20, 22–24] (OR = 0.425; 95% CI 0.217–0.912; *p* = .015) and regular dental care [22, 23, 26, 27] (OR = 0.581; 95% CI 0.298–0.946; *p* = .027) were inversely associated with dementia incidence. Since the studies conducted by Stewart et al. [24] and Stein et al. [21] enrolled only female participants, these studies were excluded from meta-regression on age. The rest of the results from gender [10, 11, 20, 22, 23, 25–28], alcohol [10, 22, 23, 26], smoking [10, 22–25, 27], depression [23, 25, 27] and denture use [20, 22, 26–28] were not associated with dementia incidence.

**Table 4:** Meta-regression analysis of association between variables and dementia incidence

Tested variables	Pooled odds ratio^a^	95% Lower limit	95% Upper limit
Age (9; 26,923) [10, 11, 21, 23–28]	1.068	1.007	1.142
Female (9; 27,823) [10, 11, 20, 22, 23, 25–28]	1.440	0.641	3.528
Alcohol (4; 10,931) [10, 22, 23, 26]	1.391	0.809	2.892
Smoking (6; 21,725) [10, 22–25, 27]	2.103	0.975	3.684
Diabetes (4; 16,466) [10, 22, 24, 25]	3.547	1.135	4.885
Hypertension (4; 5980) [22, 23, 25, 28]	3.146	1.071	4.610
Depression (3; 8322) [23, 25, 27]	1.894	0.906	3.998
High-level education attainment (4; 3502) [20, 22–24]	0.425	0.217	0.912
Denture use (5; 12,381) [20, 22, 26–28]	0.384	0.192	1.930
Regular dental care (4; 12,293) [22, 23, 26, 27]	0.581	0.298	0.946

^a^Pooled odds ratio > 1 indicates that tested variable is associated with an increase likelihood of dementia incidence

### Sensitivity analyses

The resulting estimate of the corrected OR was 0.469 (95% CI 0.307–0.718; *p* < .001) by the “trim-and-fill” method. While the corrected OR was increased, it did not change the essential thrust of pooled ORs on the effect of higher residual teeth number being associated with a lower incidence of dementia. The meta-analysis found that high residual teeth number group was robust in influencing the analysis. The results with pooled estimates of OR of dementia incidence with high residual tooth number group ranged from 0.315 to 0.740. With removal of individual studies and the lower limit of 95% CI, the results ranged from 0.277 to 0.364. The upper limits of 95% CI ranged from 0.603 to 0.792 with the removal of individual studies. The meta-regression results were confirmed even with sensitivity analyses using a second-order term.

## Discussion

### Tooth loss and dementia incidence in later life

To the best of our knowledge, the current study is the first well-designed meta-analysis that compares dementia occurrence risk among residual teeth number groups in later life. The current meta-analysis found that dementia occurrence risk in the high residual teeth number group was lowered by approximately half compared to the low residual teeth number group. However, wide variations in observation period, dementia definition, and high residual teeth group definition between studies may have led to greater heterogeneity among main findings. Despite significant dementia incidence rates between high/low residual teeth number groups as presented by pooled OR (0.483), the absolute difference of dementia incidence rates between these two groups was relatively narrow (+ 686 per 100,000 persons in low residual teeth number group). This effect can be explained by Paganini-Hill et al. [26], which had the largest number of study participants and yet had results, though with marginal significance, that ran counter to those of the meta-analysis (high residual tooth number group increased dementia incidence).

There are multiple possible mechanisms by which tooth loss can adversely affect cognitive function in later life. It has been suggested that masticatory stimulation with normal occlusion increases cerebral blood flow, activation of the cortical area, and increases levels of oxygen in blood [22, 29]. Reciprocally, poor mastication decreases orofacial sensorimotor activity, which eventually results in an overall cognitive decline [30].

### Meta-regression and factors associated with dementia in later life

The association between tooth loss and dementia occurrence may be confounded by biological and healthcare system factors. Advanced age is the strongest predictor of incidence for any type of dementia as replicated by the current analysis. This finding has been identified from an early meta-analysis, which also noted that elderly women had a higher rate of dementia incidence [31]. However, recent studies on the progression from mild cognitive impairment to Alzheimer disease present conflicting results [32, 33]. A gender difference was not identified in dementia incidence in our analysis.

A recent meta-analysis by Xu et al. [34] found a dose-response relationship between alcohol use and dementia incidence. Modest alcohol consumption was associated with a decreased risk of dementia incidence while high alcohol consumption was associated with an increased risk of dementia incidence. Data collection of alcohol intake for the analyzed studies was not quantified except for Batty et al. [10]; therefore, the lack of association between alcohol intake and dementia occurrence found in this meta-analysis could be explained by the mixed effects of modest and high-amount of alcohol consumption. Previous studies have supported that smoking is associated with dementia incidence [35, 36]. The magnitude of the relationship between smoking and dementia incidence was effaced to the point of marginal non-significance in this analysis, but potential selection bias from heterogeneous data may have occurred.

Diabetes and hypertension are cardiovascular risk factors and, when these are uncontrolled over a long-period, they may lead to atherosclerosis, which in turn may reduce cerebral blood flow. This has been the main explanation for the wide spectrum of cognitive impairment, from mild cognitive impairment to vascular and Alzheimer’s dementia [10, 37, 38].

Chewing with a removable denture is at least 30% to 40% less efficient than chewing with natural teeth [39]. Denture use might not restore the entire masticatory function in elderly patients with tooth loss as much as implants do. Therefore, denture use may not maintain cerebral blood flow in patients with tooth loss [40].

Regular dental care was associated with a decreased risk of dementia occurrence in meta-regression from 4 analyzed studies [22, 23, 26, 27]. More than half of adults in the US faced at least partial tooth loss and had not received regular dental care [41]. Except for Paganini-Hill et al. [26], which found no association between regular dental care and dementia incidence, three studies were conducted in Japan. These studies found that regular dental care reduced dementia incidence. The different lifestyle and health care systems between Paganini-Hill et al. [26] and the other three Japanese studies [22, 23, 27] may explain the different effects of regular dental care on dementia incidence [42]. For example, older Japanese adults might have more access to dental care through the Japanese Ministry of Health and Welfare initiated nationwide dental policy, the “80–20” campaign in effect since 1989 [43], as well as through a universal long-term care insurance implementation that has been happening since 2001 [44], which promotes regular dental care in later life. In a subgroup analysis, Asian studies found a greater association between tooth loss and dementia incidence while in Western studies, this association was diminished. This finding could be interpreted as possible interactions between healthcare systems (universal vs. non-universal delivery systems) and dental care access. Therefore, future comparative studies are urgently needed, to see whether a link between regular dental care and dementia incidence can be replicated in countries where different health care systems, universal vs. non-universal, have been adopted.

Education itself could create an additional reserve against clinical manifestations of dementia or educational attainment may be the result of having a greater reserve to begin with [45]. This hypothesis has been proven by a serial of cohort [46] and meta-analyses [47]. Temporal trends in developed countries, higher levels of education attainment, and better control of cardiovascular risk factors are considered likely contributors to the declining dementia risk [48, 49]. Although educational attainment is a powerful determinant of health among older adults, participation in lifelong learning, especially with a community-based approach, has a great potential to attenuate the relationship between tooth loss and dementia incidence [50]. Community-based lifelong learning programs could enhance social networks, active engagement, and encourage regular participation in intellectually stimulating activities, which may delay the progression of cognitive decline even in older adults whose cognition has already started to decline [51].

### Strengths and limitations

A strength of the current study is that the analysis was based on cohort studies which are better able to feasibly explain dementia occurrence when compared to cross-sectional studies. Another strength is the application of a structured approach to literature search and quality assessment.

There are several limitations in this study. The oral cavity, particularly in chronic periodontal disease, can affect tooth loss [20, 21, 52] and cognitive impairment [53–55] by multiple plausible explanations [7, 53]. Chronic inflammation as measured by blood inflammatory markers are, in turn, thought to play a central role in altering the inflammatory state within the brain via various microbial or cytokines activities [54, 56–59]. The mediator role of chronic periodontal disease has not been measured in the original studies, therefore, the mediator effect of chronic periodontal disease between tooth loss and dementia could not be presented in the current analysis. Heterogeneity from differing observation times, cognitive assessments, and definitions of high and low residual teeth number groups could raise concerns of overgeneralization when these findings are applied to real practice. There is potential reverse causality due to long prodromal phase of dementia. Time-person analysis might adjust an issue resulting from potential reverse causality. In addition, a time-person analysis could not be applied due to the lack of data from studies except for the most recently published study, Takeuchi et al. [22]. This analysis relied on counting residual teeth at the completion of each study. A mid-point analysis that may have provided more information of time-variable analysis could not be performed.

## Conclusions

These findings support a link between tooth loss and dementia occurrence in later life. Further studies are needed to evaluate the effects of chronic periodontitis on cognitive function in multi-point longitudinal studies. In addition, further developments in restorative dental prostheses by oral health scientists as well as optimizing lifelong community education programs for local older adults by community leaders and health policymakers may defer or reduce negative outcomes associated with tooth loss and cognitive decline.

## Additional file

Additional file 1: Supplement (DOCX 12 kb)

## Abbreviations

CI: Confidence Intervals
GRADE: Grading of recommendations assessment, development, and evaluation
OIS: Optimal information size
OR: Odds ratio
PRISMA: Preferred reporting items for systematic reviews and meta-analyses
