# Supplementary material for: Association between residual teeth number in later life and incidence of dementia: A systematic review and meta-analysis
Source: BMC Geriatr. 2018 Feb 17;18:48. doi: 10.1186/s12877-018-0729-z (PMC5816354; doi:10.1186/s12877-018-0729-z)
Supplement: Supplementary file 1 — Supplement (DOCX 12 kb) [file 12877_2018_729_MOESM1_ESM.docx]

**Supplement**

We used the following search terms to identify cohort studies of relationship between residual teeth and incidence of dementia. The search was initially performed on February 18, 2017 and finalized on March 25, 2017.

1) MEDLINE:

Search #1: (teeth [MeSH terms] OR tooth*[title/abstract] OR dental [title/abstract] OR tooth loss*[title/abstract] OR teeth loss*[title/abstract] OR missing teeth*[title/abstract] OR oral health*[title/abstract] OR dental care*[title/abstract]) AND (elderly*[title/abstract] OR later life*[title/abstract] OR older adults*[title/abstract] OR senior*[title/abstract]) AND (cognition*[title/abstract] OR cognitive impairment*[title/abstract] OR cognitive decline*[title/abstract] OR dementia*[title/abstract])

Search #2: ((cohort studies [publication type] OR observation studies [publication type] OR prospective studies [publication type]) AND adult [MeSH terms]

Search #3: (comment [publication type] OR editorial [publication type] OR guideline [publication type] OR letter [publication type] OR review [publication type] OR systematic review [publication type] OR meta-analysis [publication type])

Search #4: (#1 and #2) NOT #3

2) EMBASE

Search #1: ([title or abstract] teeth OR tooth OR dental OR tooth loss OR teeth loss or missing teeth OR oral health OR dental health OR) AND ([title or abstract] elderly OR later life OR older adults OR senior) AND ([title or abstract] cognition OR cognitive impairment OR cognitive decline OR dementia)

Search #2: ([publication type] editorial OR letter OR review OR note) OR ([evidence based medicine] systematic review OR meta analysis)

Search #3: (#1 and #2) NOT #3
